# Docking Simulation and Sandwich Assay for Aptamer-Based Botulinum Neurotoxin Type C Detection

**DOI:** 10.3390/bios10080098

**Published:** 2020-08-12

**Authors:** In-Hwan Oh, Dae-Young Park, Ji-Man Cha, Woo-Ri Shin, Ji Hun Kim, Sun Chang Kim, Byung-Kwan Cho, Ji-Young Ahn, Yang-Hoon Kim

**Affiliations:** 1 School of Biological Sciences, Chungbuk National University, 1 Chungdae-Ro, Seowon-Gu, Cheongju 28644, Korea; etoneru@chungbuk.ac.kr (I.-H.O.); daepark4698@nate.com (D.-Y.P.); 28904865@daum.net (J.-M.C.); wr1203@chungbuk.ac.kr (W.-R.S.); 2 Department of Biological Sciences, Korea Advanced Institute of Science and Technology, 291 Daehak-ro, Yuseong-gu, Daejeon 34141, Korea; kjhwlgns0997@kaist.ac.kr (J.H.K.); sunkim@kaist.ac.kr (S.C.K.); bcho@kaist.ac.kr (B.-K.C.)

**Keywords:** Botulinum neurotoxin type C, aptamer, docking simulation, aptamer-based sandwich assay

## Abstract

Aptamers are biomaterials that bind to a target molecule through a unique structure, and have high applicability in the diagnostic and medical fields. To effectively utilize aptamers, it is important to analyze the structure of the aptamer binding to the target molecule; however, there are difficulties in experimentally identifying this structure. In the modern pharmaceutical industry, computer-driven docking simulations that predict intermolecular binding models are used to select candidates that effectively bind target molecules. Botulinum toxin (BoNT) is the most poisonous neurotoxin produced from the *Clostridium botulinum* bacteria, and BoNT/C, one of the eight serotypes, causes paralysis in livestock. In this study, the aptamers that bound to BoNT/C were screened via the systematic evolution of ligands by exponential enrichment, and the binding affinity analysis and binding model were evaluated to select optimal aptamers. Based on surface plasmon resonance analysis and molecular operating environment docking simulation, a pair of aptamers that had high binding affinity to BoNT/C and were bound to different BoNT/C sites were selected. A sandwich assay based on this aptamer pair detected the BoNT/C protein to a concentration as low as ~0.2 ng Ml^−1^. These results show that docking simulations are a useful strategy for screening aptamers that bind to specific targets.

## 1. Introduction

Botulism is a neuromuscular disease resulting from toxins produced by *Clostridium botulinum* bacteria [1]. These bacteria produce botulinum neurotoxins (BoNTs) that are approximately 150 kDa, and are composed of a protein complex (heavy chain—nontoxic/light chain—toxic) linked together by disulfide bonds [2]. The heavy chain affects binding to specific receptors, such as the vesicular proteins synaptic vesicle protein 2 and synaptotagmin, and binds presynaptically to neuronal cells [3]. After that, the BoNT/receptor complex, including the light chain, is internalized into neuronal cells via endocytosis [4]. Once inside the neuronal cell, the light chain is translocated into the cytosol upon acidification of vesicles. The zinc-endopeptidase cleaves synaptic proteins, such as the vesicle-associated membrane protein synaptobrevin, synaptosome associated protein 25, and syntaxin [5]. These proteins play an important role in the fusion of synaptic vesicles at release sites [6]. The BoNTs specifically inhibit the release of vesicular neurotransmitter as a “blocking factor” to prevent the release of certain neurotransmitters.

Among the eight known serotypes of BoNTs (A to FA), BoNT serotypes C and D corrupt animals and birds, and potentially affect humans [7]. Recently, botulism by BoNT/C has become an emerging and serious problem worldwide [8], notably in livestock, where it causes a high mortality rate and economic losses. Primary diagnosis of botulism is generally based on clinical symptoms, but since the symptoms are not specific, laboratory confirmation is required to determine the BoNT serotype. The mouse bioassay, a representative method utilized for the identification of botulism, demonstrates the presence of BoNT or BoNT-producing Clostridia in samples collected from symptomatic animals, feed, or the environment [9]. Other tests detect an antibody response in an animal affected by botulism. However, these assays are expensive [10], time-consuming [11], labor-intensive [12], and involve ethical concerns with regard to the use of animals [13]. Alternative biological techniques that are rapid, specific, and involve reliable capture materials are urgently needed to confirm botulism [14].

Aptamers are short single-stranded DNA or RNA oligonucleotides that bind to diverse targets, such as proteins, metal ions, nucleic acids, cells, small molecules and microorganisms, and are also known as chemical antibodies [15]. Aptamers naturally fold at room temperature into different three-dimensional structures and are generated against a specific target using an iterative approach termed systematic evolution of ligands by exponential enrichment (SELEX) [16]. The three-dimensional structure of aptamers allows binding to specific targets with specificity and high affinity [17]. In addition, easy chemical modifications, such as 5′ amine modification and Cy5-labeling, as well as the stability of aptamers, make them attractive agents [18]. Due to these advantages, aptamers are used in various fields, including imaging, drug delivery, and as biosensors [19,20,21,22].

In the pharmaceutical industry, molecular docking is the most frequently used method to identify ligands, because it predicts ligand-target interactions at the molecular level [23]. In general, there are several difficulties in experimentally verifying the interaction of a protein with a ligand, but docking simulation is a more effective approach to identify a ligand that binds to a specific site of a target substance because it is relatively simple to access [24]. As computer science evolved, various molecular docking simulation programs were introduced, and based on this, research was conducted to predict unknown complex structures [25]. These studies also include predicting the complex of aptamers and target substances [26]. Aptamer docking simulation is utilized not only in understanding how aptamers are bound to targets [27], but also to make aptamers more economical and effective by reducing unnecessary nucleotides of aptamers [28]. Therefore, docking simulation serves as a useful strategy not only for the additional modification process of aptamers, but also at the screening stage of aptamers. In the present study, BoNT/C-specific binding ssDNA aptamers were generated by the SELEX process, an optimal aptamer pair was selected based on the analysis of the binding affinity through surface plasmon resonance (SPR) and the prediction of the binding model with BoNT/C through a molecular docking simulation. The selected aptamers quantitatively detect target proteins in sandwich assays.

## 2. Materials and Methods

### 2.1. In Vitro Selection of DNA Aptamers to BoNT/C

The 100 nucleotide single-stranded DNA (ssDNA) library contained 40 nucleotides of randomized sequence and defined primer binding sites at both ends (5′-GGT AAT ACG ACT CAC TAT AGG GAG ATA CCA GCT TAT TCA ATT (42 mer)-random sequence (40 mer)-AGA TTG CAC TTA CTA TCT (18 mer)-3′). The library was used as an initial ssDNA library and amplified by polymerase chain reaction (PCR). For PCR amplification, a 5′ forward primer and a 3′ biotin-modified reverse primer were used to obtain double-stranded DNA (dsDNA). A 20 cycle PCR amplification was performed in a 50 μL volume of a mixture containing DNA template, 10× buffer, dNTPs, each primer, and Ex Taq polymerase (Takara, Shiga, Japan). PCR products were purified using a QIA quick PCR purification kit (Qiagen, Germantown, MD, USA). After purification, the reaction was added to 50 μL streptavidin agarose resin (Thermo scientific, Waltham, MA, USA) and incubated at room temperature (RT) for 1 h with gentle shaking. The biotinylated DNA strand bound to streptavidin agarose resin was removed by centrifugation (13,000 rpm, 4 °C, 10 min) and the ssDNA in the upper layer was recovered.

Isolated ssDNA was purified by phenol/chloroform extraction and ethanol precipitation, and precipitated ssDNA was then resuspended in a suitable volume of distilled water. For each round of SELEX, 50 μL ssDNA (323.1 pmol) was added to 50 μL 2× PBS. The mixture was then heated at 85 °C and allowed to slowly form structures at RT. Structured DNA aptamers were incubated with his-tagged recombinant C. botulinum BoNT-C1 light chain (21.54 pmol) (R&D Systems, Minneapolis, MN, USA) at 4 °C overnight in 1× PBS. The aptamer-BoNT/C complex was fixed by performing a reaction between the his-tag at the end of the protein and nickel nitrilotriacetic acid (Ni-NTA) agarose resin (Qiagen, Germantown, MD, USA) at 4 °C for 1 h. The aptamer-BoNT/C complexes immobilized on Ni-NTA agarose resin were washed five times with a wash buffer (60 mM imidazole, 500 mM NaCl, and 20 mM Tris-HCl (pH 7.9)), to remove nonspecific aptamers. BoNT/C bound aptamers were then eluted with 200 μL 1× PBS at 85 °C for 20 min. BoNT/C bound aptamers from the eluted BoNT/C-aptamer complexes were purified by phenol/chloroform extraction and ethanol precipitation. The eluted BoNT/C aptamer pools were used as a template for the next round of SELEX. A total of 12 rounds of selection were performed; negative selection was performed after 6 rounds to remove aptamers with nonspecific binding. Negative selection was performed by reacting the aptamer with a Ni-NTA agarose resin at 4 °C for 1 h, and only the unbound upper layer was recovered to remove any aptamers binding to the Ni-NTA agarose resin. The eluted BoNT/C aptamer pools from negative selection were used as a template for the next round of SELEX.

### 2.2. Identification of the Optimal Selection Round and Screening of Aptamer Candidates

To select a suitable round for screening aptamer candidates, the concentration of BoNT/C aptamer pools eluted from each round was estimated using a nanodrop spectrophotometer (Thermo, Waltham, MA, USA) at an absorbance of 260 nm. In addition, real-time PCR was performed with eluted BoNT/C aptamer pools from rounds 9–12 and the iQTM SYBR Green Supermix kit (Bio-Rad, Santa Rosa, CA, USA), using a MiniOpticon real-ime PCR fluorescence signal detection system (Bio-Rad, Santa Rosa, CA, USA). Reactions were performed in a 20 μL volume containing 2 μL of eluted aptamers, 0.5 μL forward primer, 0.5 μL reverse primer (100 nM each), 10 μL iQTM SYBR Green Supermix and 7 μL distilled water. After pre-denaturation at 95 °C for 5 min, 40 cycles of amplification were performed at 95 °C for 20 s, 55 °C for 20 s, 72 °C for 20 s, and a final extension at 72 °C for 5 min. Analysis of real-time PCR was conducted by the CFX manager software program (Bio-Rad, Santa Rosa, CA, USA). All analyses using the elution pool were conducted three times.

Cloning was performed for sequencing using the selected round 10 aptamer pool and the T-BluntTM PCR Cloning kit (Solgent, Daejeon, Korea). Clone acquisition and aptamer sequence analysis were performed using conventional methods [29].

### 2.3. Affinity Test for BoNT/C Bound Aptamers (SPR)

An aptamer binding affinity test was performed using the BIAcore 3000 (BIAcore^®^ AB, Uppsala, Sweden), with a CM5 sensor chip (GE Healthcare, Buckinghamshire, UK). To immobilize BoNT/C to the CM5 sensor chip, BoNT/C was activated with 100 μL of a 1:1 mixture of 0.05 M N-hydroxysuccinimide (NHS; GE Healthcare, Uppsala, Sweden) and 0.2 M N-ethyl-N’-dimethylaminopropyl carbodiimide (EDC; GE Healthcare, Uppsala, Sweden), by modifying the carboxymethyl groups of dextran. BoNT/C was diluted 1:100 in 10 mM sodium acetate (pH 4.5) and then injected five times into the sensor, until it was no longer immobilized on the sensor surface of flow cell-2 [30]. Flow cell-1 was treated in the same way, but without BoNT/C. The remaining activated dextran was blocked with 1 M ethanolamine hydrochloride (pH 8.5). The flow rate was maintained at 10 μL min^−1^ throughout the experiment, and HEPES buffered saline (HBS)-EP buffer (10 mM HEPES pH 7.4, 150 mM NaCl, 3 mM EDTA, and 0.005% polysorbate 20 *v*/*v*) was used as the running buffer. Each aptamer candidate was diluted in running buffer at various concentrations from 300–700 nM, and sequentially injected over the sensor surface of flow cell 1 and 2 for 1 min. Following each binding step, the surface of the sensor chip was regenerated with 1 M NaCl, 50 mM NaOH until the signal reached baseline levels. The dissociation constants (K*_D_*) of the aptamer-BoNT/C complexes were determined using the BIAevaluation 4.1 software (Biacore AB, Uppsala, Sweden). All analyses of aptamer affinity were performed in triplicate.

### 2.4. Molecular Operating Environment (MOE)-Docking Simulation of Aptamers Bound to BoNT/C

To select the aptamers suitable for the sandwich assay method, a docking simulation of three aptamers (BoNT/C 10, 12, 14) with high binding ability to BoNT/C was performed. To compare the sites where aptamers bound to the target protein through the docking simulation, PDB format files containing information about the structure of the aptamer and protein were obtained. The process of acquiring the PDB file for the aptamers was performed through the referenced paper [29]. The X-ray structure of BoNT/C (PDB code-2QN0) was obtained from the RCSB Protein Data Bank site (https://www.rcsb.org/), and used as a template to predict the binding sites of three aptamers (BoNT/C 10, 12, 14).

Based on the structure of the aptamer candidates and BoNT/C, a docking simulation was conducted using the MOE program (MOE version 2016.0802) with the triangle matcher method. Prior to the docking simulation, the energies of aptamers and BoNT/C were minimized with MOE using the AMBER10 force field until the root mean square (RMS) gradient of the potential energy was less than 0.1 kJ mol^−1^ Å^−1^. The predicted aptamer candidates-BoNT/C docking simulation results were extracted into a PDB file. The binding site of the aptamer was analyzed through PyMOL (1.1 eval) and Ligplot+ (v2.2) software.

### 2.5. BoNT/C Detection using An Aptamer-Based Sandwich Assay

To evaluate the specificity of the aptamers by the sandwich assay method, each aptamer (BoNT/C 10, BoNT/C 14) was labeled with an amine and Cy5 at the 5’ end (Bioneer, Daejeon, Korea). Different botulinum neurotoxin serotypes and other proteins present in the blood, such as botulinum neurotoxin type E and bovine serum albumin (BSA), were used as negative controls. The 1 μM 5′ amine-modified aptamers (BoNT/C 14) dissolved in the 1× PBS were formed at 85 °C for 5 min and cooled at RT for 2 h. The aptamer that formed the structure was added to wells of a DNA-BIND (Amine binding) 96-well plate (Costar, New York, NY, USA) and incubated for 1 h. One hundred microliters of 100 nM protein (BSA, BoNT/E) and 1× PBS containing 0.1 to 1 ng mL^−1^ of BoNT/C were then added to BoNT/C 14-coated wells in a 96-well plate and incubated for 1 h at 4 °C. Cy5-labeled aptamer (1 μM BoNT/C 10) dissolved in the 1× PBS was added to each well and incubated for 30 min at RT. After each step, 100 µL distilled water was added to each well to remove unbound aptamers and proteins. The absorbance of each well was measured at an excitation of 646 nm and an emission of 662 nm, using a SpectraMax M2 Multi-Mode microplate reader (Molecular Devices, San Jose, CA, USA). Analysis through an aptamer-based sandwich assay was conducted three times.

## 3. Results

The SELEX process was applied to BoNT/C and ssDNA aptamers from 100 nucleotides of random sequence. Amplification of a chemically synthesized random library (about 10^16^ diverse sequences) was performed by PCR, and then ssDNA was generated by removal of the biotinylated strand using streptavidin agarose resin. The ssDNA molecules were bound to BoNT/C at a protein to DNA molar ratio of 1:15. Finally, BoNT/C binding ssDNA molecules from the eluted BoNT/C-ssDNA complex were applied to Ni-NTA agarose resin and elution buffer. The eluted ssDNA molecules were used as a template for the next round of SELEX. A total of 12 cycles of the SELEX process were conducted for enrichment of high-affinity sequences. Negative selection was employed to remove nonspecific bound aptamers. When the aptamer pool amplified in each step was compared by electrophoresis, it was confirmed that it was amplified to a suitable size, and through this, it was determined that SELEX proceeded without abnormality.

**Figure 1:**
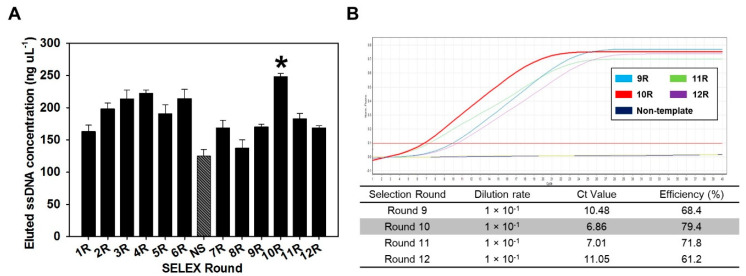
Measurement of eluted ssDNA concentration and the dynamic range of a real-time polymerase chain reaction (PCR) assay for selection of the optimal round. (**A**) ssDNA of each round, obtained through the systematic evolution of ligands by exponential enrichment (SELEX) procedure. Negative selection (NS) was performed after round six to defend enrichment of botulinum toxin type C (BoNT/C) nonspecific binding aptamers. Eluted ssDNA pools from round 10 are indicated by a high concentration (*). (**B**) Amplification of 10-fold serially diluted aptamer from each round. The signal was detected with SYBR^®^ Green I dye at an excitation of 494 nm and an emission of 521 nm. Dilution rate refers to the dilution ratio of the elution pool of each round used as a template for real-time PCR. The Ct value refers to the number of amplification cycles required to reach a fixed signal threshold.

After negative selection, there was a tendency to increase the concentration of the BoNT/C aptamer pool from round 8 (137.1 ng µL^−1^) to round 10 (248.1 ng µL^−1^), but it was confirmed that the concentration decreased after round 10 (Figure 1A). The tendency that the concentration of aptamer increased and then decreased again suggests that after negative selection, the BoNT/C-specific aptamer gradually accumulated, and then the concentration decreased, due to a decrease of the nonspecific aptamers. These results indicated that the aptamer pool eluted from 10 rounds was composed of a high proportion of aptamers that specifically bound to BoNT/C.

In addition, the eluted BoNT/C aptamer pool after negative selection was analyzed by real-time PCR. The eluted BoNT/C aptamer pool from rounds 9–12 was serially diluted 10 times to 10^−2^, and used as a template for real-time PCR. Representatively, the measurement results of Ct values for rounds 9, 10, 11, and 12 are 10.48, 6.86, 7.01, and 11.05, respectively (Figure 1B). These results implied that the aptamer was present in a high concentration in the aptamer pool recovered in 10 rounds. By comparing the elution concentration of each round and the Ct value of real-time PCR, round 10 was selected as the optimal round to screen aptamer candidates. Based on the results, the aptamer pool eluted in 10 rounds was used as a template for sequencing, and 15 aptamer candidates with different random sequences were obtained (Supplementary Table S1). The lengths of the aptamer sequences were confirmed to be similar overall, indicating that the amplification process was performed smoothly in the overall SELEX process.

The binding affinities of the selected 15 aptamer candidates were analyzed on a SPR-based Biacore 3000 instrument, as discussed in Materials and Methods. The technique has been used to measure the association (K_on_) and disassociation (K_off_) of two molecules, such as protein-protein or protein-DNA interactions, on a sensor chip surface [31]. The quotient of these rates (K_off_/K_on_) is explained as the equilibrium-dissociation constant (K*_D_*) of two molecules. The dissociation constant (K*_D_*) for the interaction of each aptamer with BoNT/C was determined using the BIA-evaluation software, version 4.1. The selected aptamers had K*_D_* values ranging from 4.28 × 10^−8^ M to 6.89 × 10^−10^ M. These comprehensive results were obtained using the BIA-evaluation software (Supplementary Table S1). Most aptamers isolated through SELEX had K*_D_* values of nanomolar affinity. This suggests that each step of the SELEX process was performed appropriately and only aptamers with high affinity for BoNT/C remained.

For the analysis of the binding model, the BoNT/C 10, BoNT/C 12 and BoNT 14 aptamers with high binding affinity to BoNT/C were selected, and the binding model of three aptamers with BoNT/C was analyzed through the MOE program (MOE version 2016.0802). The binding sites of aptamers were checked through PyMOL (1.1 eval) and Ligplot+ (v2.2) software (Table 1). The three possible aptamer pairs (BoNT/C 10–12, 10–14, 12–14) were compared, and it was confirmed that when BoNT/C 12 and 14 were used in a pair, the binding sites of the aptamers overlapped (Table 1 (*)). Using aptamers with the same binding site is unsuitable for sandwich assay, because it may result in a decrease in binding ability to the target protein. In the other two cases (BoNT/C 10–12, 10–14), the binding affinity between the three aptamers was compared to select the optimal aptamer pair. BoNT/C 14 had the highest binding affinity, and BoNT/C 10 had the next highest binding affinity (Supplementary Table S1). Therefore, it was expected that BoNT/C was detected more effectively using BoNT/C 10, 14 in pairs, than by using BoNT/C 12 with relatively low binding affinity.

**Figure 2:**
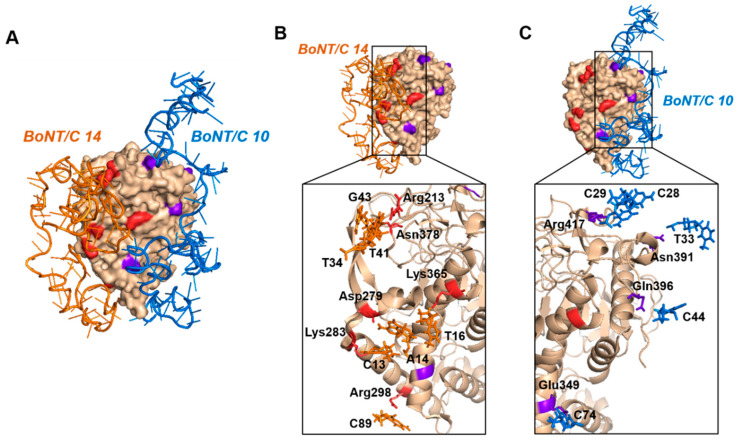
Predicted binding sites of the botulinum toxin type C (BoNT/C) and BoNT/C 10 (blue), BoNT/C 14 (orange) aptamer complex. The BoNT/C 10 binding site is shown in purple; the BoNT/C 14 binding site is shown in red. (**A**) A complex of aptamers (BoNT/C 10, BoNT/C 14) bound to BoNT/C; (**B**) residues that form the aptamer (BoNT/C 14) and protein (BoNT/C) binding site; C) residues that form the aptamer (BoNT/C 10) and protein (BoNT/C) binding site.

Based on the above results, two aptamers (BoNT/C 10, 14) having high binding affinity to BoNT/C and having different binding regions were selected as a pair. When the selected aptamer pair was combined with BoNT/C, it was predicted that there was no collision region between the aptamer regions that were not associated with BoNT/C (Figure 2). In addition, when confirming the binding site of the aptamer pair to BoNT/C, it was confirmed that a number of hydrogen bonds existed. These results indicated that when BoNT/C 10, 14 were used as a pair, they retained the ability to bind to BoNT/C in different regions and could be utilized in a sandwich assay.

**Figure 3:**
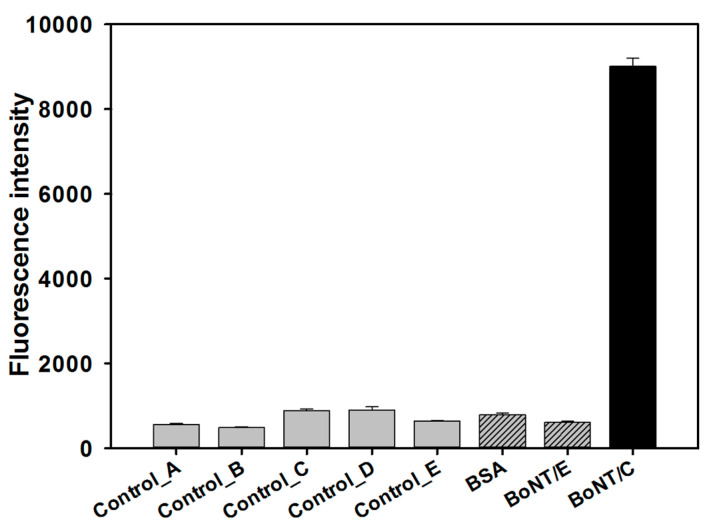
Specificity test of the selected aptamer pair to botulinum toxin type C (BoNT/C) by aptamer-based sandwich assay. Control_A: signal of the plate surface; Control_B: signal on the surface where the aptamer (BoNT/C 14 without Cy5 labelling) is attached alone without a protein sample; Control_C: signal of the Cy5-labeled aptamer (BoNT/C 10) alone without a protein sample; Control_D: signal of the aptamer pair (Cy5-labeled BoNT/C 10, BoNT/C 14 without Cy5 labelling) without protein sample; Control_E: signals of the protein sample (BoNT/C).

Modification was performed to utilize the selected aptamers based on SPR analysis and docking simulation as a sandwich assay. BoNT/C 14, which had the highest binding affinity to the target protein, was modified by an amine group at the 5’ end, and was immobilized on the surface of the 96-well plate to capture the target protein. BoNT/C 10, which was predicted to bind to a different region from BoNT/C 14, was modified by Cy5 at the 5’ end to obtain fluorescence intensity. In addition to the target protein, BSA and BoNT/E were used as negative controls, and the Cy5 signal in the 96-well plate was measured through the fluorescence of Cy5-labelled BoNT/C 10. As shown in Figure 3, the fluorescence intensity of the well with added BoNT/C is high, and the fluorescence intensity of the well with the negative control is low. BoNT/E is one of the serotypes that induces botulism in humans and animals, and shows a fluorescence intensity similar to that of other control, BSA. This result suggested that the selected aptamer pair (BoNT/C 10, 14) specifically bound to BoNT/C and to different sites of the protein.

**Figure 4:**
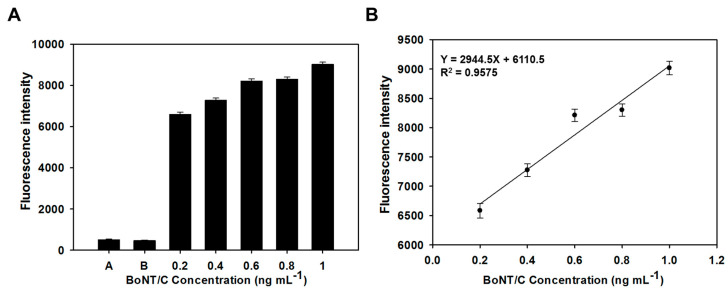
Detecting ability test of botulinum toxin type C (BoNT/C)-specific binding ssDNA aptamers based on sandwich assay. (**A**) Fluorescent intensity of the aptamer-based sandwich assay; A: Signal of the plate surface, B: Signal of the aptamer pair (Cy5-labeled BoNT/C 10, BoNT/C 14 without Cy5 labelling) without BoNT/C. (**B**) Linear response to a variety of BoNT/C concentrations.

In addition, since BoNT/C 10, 14 had a high specificity for BoNT/C, it confirmed the possibility of selectively detecting BoNT/C, even in an environment in which multiple proteins were mixed. Fluorescence sensitivity to different concentrations of BoNT/C was evaluated by an aptamer-based sandwich assay. When the concentration of BoNT/C ranged from 0.2 to 1.0 ng mL^−1^, the fluorescence intensity values increased as the concentration of BoNT/C increased; fluorescence was not detected at a concentration of BoNT/C lower than 0.2 ng mL^−1^ (Figure 4A). These results showed that the fluorescence signal varied depending on the amount of BoNT/C present in the sample. In addition, a formula was obtained by plotting the fluorescence intensity according to the concentration of BoNT/C, and a linear response was acquired in the range of BoNT/C 0.2 to 1.0 ng mL^−1^ (Figure 4B). These results demonstrate that the aptamers selected through docking simulation effectively bound to different parts of BoNT/C, so that low concentrations of BoNT/C could be quantitatively detected.

## 4. Conclusions

In the pharmaceutical and biotechnology fields, various studies have been conducted to obtain materials that bind to specific regions of target substances and analyze binding regions. It is difficult to identify the binding region by analyzing the actual structure, so a different approach is required. Docking simulation is a computer simulation tool used to model the interaction between two molecules. This method is widely used in the pharmaceutical and biotechnology fields to develop drug candidates that bind to specific active sites of a target substance. Aptamers are attracting attention in various fields because they bind to target substances based on their unique structure. In this study, two BoNT/C-specific aptamers (BoNT/C 10, 14) were selected through evaluation of binding affinity via SPR analysis and identification of binding sites through docking simulation. The selected aptamers formed hydrogen bonds at different parts of BoNT/C, and there were no areas of collision between the aptamers. The sandwich assay incorporating the two aptamers selectively detected BoNT/C, and was able to detect concentrations down to at least 0.2 ng mL^−1^. These results indicated that the two selected aptamers were bound to different parts of BoNT/C, and that docking simulation was a useful strategy for screening aptamers that bind to specific regions. Furthermore, docking simulation selected aptamers that bound to the active site of the target substance, and the selected aptamers could function as neutralizing and therapeutic agents, as well as diagnostic probes.

## Figures and Tables

**Table 1:** Binding site of BoNT/C and aptamer complex predicted by MOC 2016.0803 program.

BoNT/C 10		BoNT/C 12		BoNT/C 14	
Aptamer	BoNT/C	Aptamer	BoNT/C	Aptamer	BoNT/C
C28	Arg417	T34 *	Lys283,Lys287	C13 *	Lys283
C29	Arg417	A35 *	Lys283,Gln356	A14	Asp279
T33	Asn391	T37	Lys287	T16	Lys365
C44	Gln396	T57	Lys346	T34	Arg213
C74	Glu349	G78	Thr309	T41	Arg213
				G43	Asn378
				C89	Arg298

## Supplementary Materials

The following are available online at https://www.mdpi.com/2079-6374/10/8/98/s1, Table S1. List of aptamer candidates isolated for BoNT/C and assessment of their binding affinity by SPR assay.
