# Supplementary material for: Docking Simulation and Sandwich Assay for Aptamer-Based Botulinum Neurotoxin Type C Detection
Source: Biosensors (Basel). 2020 Aug 12;10(8):98. doi: 10.3390/bios10080098 (PMC7460441; doi:10.3390/bios10080098)
Supplement: Supplementary file 1 [file biosensors-10-00098-s001.pdf]

biosensors

MDPI

Article

Docking Simulation and Sandwich Assay for Aptamer-Based Botulinum Neurotoxin Type C Detection

In-Hwan Oh [1,†], Dae-Young Park [1,†], Ji-Man Cha [1,†], Woo-Ri Shin [1], Ji-Young Ahn [1,*], Yang-Hoon Kim [1,*], Ji Hun Kim [2], Sun Chang Kim [2] and Byung-Kwan Cho [2]

1 School of Biological Sciences, Chungbuk National University, 1 Chungdae-Ro, Seowon-Gu, Cheongju 28644, Korea; etoneru@chungbuk.ac.kr (I.-H.O.); daepark4698@nate.com (D.-Y.P.); 28904865@daum.net (J.-M.C.); wr1203@chungbuk.ac.kr (W.-R.S.)
2 Department of Biological Sciences, Korea Advanced Institute of Science and Technology, 291 Daehak-ro, Yuseong-gu, Daejeon 34141, Korea; kjhwlgns0997@kaist.ac.kr (J.H.K); sunkim@kaist.ac.kr (S.C.K.); bcho@kaist.ac.kr (B.-K.C.)
* Correspondence: jyahn@chungbuk.ac.kr (J.-Y.A.); kyh@chungbuk.ac.kr (Y.-H.K.); Tel.: +82-43-261-2301 (J.-Y.A.); Tel.: +82-43-261-3575 (Y.-H.K.)
† These authors contributed equally to this work.

Received: 22 July 2020; Accepted: 10 August 2020; Published: 12 August 2020

[Supporting Information]

Docking Simulation and Sandwich assay for Aptamer-based Botulinum Neurotoxin Type C Detection

Supplementary Table S1. List of sequences of aptamer candidates isolated for BoNT/C and assessment of their binding affinity by SPR assay

Name	Aptamer sequence (N40)	Affinity (K_D, M)	Size (bp)
BoNT/C 1	GGGTGGTGGTACCTACCTTGAGCGAGTCGAGATCACGCTG	9.98×10^{-9}	40
BoNT/C 2	GCGGGGTTTGTGCCATCCTGTGCCATTGAATGTTGGGGCG	8.05×10^{-9}	40
BoNT/C 3	GCCGCGTCGGTGGCGATTGTGTATGCGGTTGTCCCCGGGG	1.08×10^{-8}	40
BoNT/C 4	TGGTGAGTATAACCCTAGAGGCTTCGGCTTTGAGTCGGGG	1.37×10^{-8}	40
BoNT/C 5	GGTAAGCTGTCTCGTGGGTCGTGTTAGGGGCGCGTCTCGG	2.17×10^{-8}	40
BoNT/C 6	GCGTGGTGGCGAAGTTTAGTATATCGTATCTTGCCTAGGG	7.30×10^{-9}	40
BoNT/C 7	CAGCGGCCCTACAGTGCGTATTGATGGCCTTTCTGCTGGG	3.93×10^{-8}	40
BoNT/C 8	CCTTAGTGCGGTACGTGACTATTGTTGAATATGCGCTGGG	4.28×10^{-8}	40
BoNT/C 9	CCGGTATCACACTGTTGGAAGGGGCGTATTTACTCTCGGCA	3.96×10^{-8}	41
BoNT/C 10	GCAAAGGTTTTGAATAACCGCTGAGCTCTCCCGTTCTGCG	1.55×10^{-9}	40
BoNT/C 11	GGCGTATCTCCTCACCTATGTCAGTCGTGTTGCCGGTTCG	2.39×10^{-8}	40
BoNT/C 12	TGGTGGTCAGTGAGTGGTCGGGTCTCTGCATCTCTGTGTG	4.20×10^{-9}	40
BoNT/C 13	CGGATGCGGGTAATTGAATTAGTACGGTCTGCCCCTTGTG	1.06×10^{-8}	40
BoNT/C 14	GGATCTGCTGTGTACCTCGCTGCTCGACTGGACTAGTCCG	6.89×10^{-10}	40
BoNT/C 15	CGGATGCGGGTAATTGAATTAGTACGGTTTGCCCCTTGTG	3.16×10^{-8}	40
